# The efficacy of a behavioral activation intervention among depressed US Latinos with limited English language proficiency: study protocol for a randomized controlled trial

**DOI:** 10.1186/1745-6215-15-231

**Published:** 2014-06-18

**Authors:** Anahi Collado, Katherine E Long, Laura MacPherson, Carl W Lejuez

**Affiliations:** 1 Center for Addictions, Personality, and Emotion Research (CAPER), 2103 Cole Field House, University of Maryland, College Park, MD 20742, USA

**Keywords:** Behavioral activation, Randomized controlled trial, Latinos, Depression, Spanish-speaking

## Abstract

**Background:**

Major depressive disorder is highly prevalent among Latinos with limited English language proficiency in the United States. Although major depressive disorder is highly treatable, barriers to depression treatment have historically prevented Latinos with limited English language proficiency from accessing effective interventions. The project seeks to evaluate the efficacy of behavioral activation treatment for depression, an empirically supported treatment for depression, as an intervention that may address some of the disparities surrounding the receipt of efficacious mental health care for this population.

**Methods/design:**

Following a pilot study of behavioral activation treatment for depression with 10 participants which yielded very promising results, the current study is a randomized control trial testing behavioral activation treatment for depression versus a supportive counseling treatment for depression. We are in the process of recruiting 60 Latinos with limited English language proficiency meeting criteria for major depressive disorder according to the Diagnostic and Statistical Manual of Mental Disorders 4^th^ and 5^th^ Edition for participation in a single-center efficacy trial. Participants are randomized to receive 10 sessions of behavioral activation treatment for depression (*n* = 30) or 10 sessions of supportive counseling (*n* = 30). Assessments occur prior to each session and at 1 month after completing treatment. Intervention targets include depressive symptomatology and the proposed mechanisms of behavioral activation treatment for depression: activity level and environmental reward. We will also examine other factors related to treatment outcome such as treatment adherence, treatment satisfaction, and therapeutic alliance.

**Discussion:**

This randomized controlled trial will allow us to determine the efficacy of behavioral activation treatment for depression in a fast-growing, yet highly underserved population in US mental health services. The study is also among the first to examine the effect of the proposed mechanisms of change of behavioral activation treatment for depression (that is, activity level and environmental reward) on depression over time. To our knowledge, this is the first randomized controlled trial to compare an empirical-supported treatment to a control supportive counseling condition in a sample of depressed, Spanish-speaking Latinos in the United States.

**Trial registration:**

Clinical Trials Register: NCT01958840; registered 8 October 2013.

## Background

The lifetime prevalence rate of major depressive disorder (MDD) in the general US population is approximately 16.6% [1]. Among Latinos in the US, MDD rates range from 3% to 18% [2-4]. However, in primary care settings, MDD rates are reportedly as high as 25% among Latinos with limited English language proficiency (LEP) [5-8]. Considering that Latinos constitute the largest US minority group, and approximately 50% have LEP, elevated rates of depression among Latinos with LEP represents a great public health concern [9]. The strikingly high rates of MDD among Latinos with LEP may stem from language barriers that promote social isolation and limited access to health care, which in turn result in distress and low perceived self-efficacy [10,11]. Altogether, the elevated rates of depression among Latinos with LEP and the growing numbers of this population in the US point to a pressing need for efficacious treatments for depression for this group.

Although MDD is generally highly treatable, many barriers preclude Latinos with LEP from accessing quality mental health care [12,13]. In general, less than 5% of Latino immigrants afflicted with psychological disorders seek mental health services from specialized practitioners [14] due to a combination of linguistic and economic barriers, cultural stigma, and the lack of empirically supported treatments for this population [13-15]. Moreover, this group’s rates of treatment attrition are significantly higher than for non-Latino White-Americans [16,17]. Historically, the aforementioned barriers have resulted in Latinos being underserved and under-represented in both research and clinical trials.

Despite the barriers to receiving treatment, Latinos with LEP tend to express positive attitudes towards psychosocial treatments for depression [18,19]. This finding is consistent with a valued belief among Latinos of doing one’s part in one’s recovery (“poner de su parte”) rather than relying on medication which is viewed as more passive [15,20,21]. The concept of *poner de su parte* is especially consistent with the framework of behavioral psychotherapies [22]. In fact, earlier research regarded behaviorally focused psychotherapies to be appropriate treatments for Latinos with mental health needs [23]. Specifically, behavioral therapies may allow Latino clients to perceive control over everyday situations, in stark contrast to the lack of control they experience resultant from widespread socioeconomic marginalization, including issues related to poverty and racism [23].

Support for behavioral treatments in Latinos was first provided three decades ago [23]. In this study, group format behavioral therapy and group format cognitive therapy demonstrated superior results relative to the wait-list control group among Puerto Rican women. However, at a 5-week post-treatment follow-up assessment, treatment gains were only maintained for those assigned to behavioral therapy. More recently, behavioral activation (BA) has been shown to be a promising form of behavior therapy for Latinos with LEP. According to BA, depression results when constructive (that is, non-depressive) behaviors are not positively reinforced. The depressive state is maintained by negative reinforcement, such as avoidance and receiving sympathy from others [24]. Through BA, individuals engage in activities that are important and enjoyable to them [25]. Concurrently, participants are required to monitor their mood while they engage in these activities. This increases their awareness of the effect that behaviors have over their mood [25,26]. To date, four meta-analyses have revealed the effectiveness of the BA approach [27-30]. Despite the empirical support of BA, only two different open-label studies thus far have demonstrated the promise of delivering BA in Latino populations [31,32].

The first open-trail pilot study (*n* = 10) evaluated a culturally modified version of BA in Spanish [31] (modified from [33]). In addition to scheduling pleasant activities, this BA version incorporated cognitive rehearsal, skill building, mindfulness, exposure to activities for which avoidance is displayed, and role-playing. As part of the cultural modifications, the investigators indicated simplifying the treatment rationale and paying close attention to values commonly attributed to Latinos that would affect the course of treatment. Treatment outcomes suggested significant decreases in depression severity in the intent-to-treat sample. However, despite the promising results, only three clients completed the 12-session treatment.

The second pilot study evaluated an alternative BA version: the behavioral activation treatment for depression (BATD) [26,34]. BATD has been described as both brief and efficient [35,36]. Furthermore, the ideographic nature of BATD facilitates the incorporation of unique personal values of individuals throughout treatment which is especially important given the high degree of within-group heterogeneity among Latinos [37]. In randomized controlled trials (RCTs) with underserved minority samples facing similar problematic retention rates and treatment access barriers as US Latinos, those who were randomized to a BA were less likely to drop-out relative to those assigned to treatment as usual [38-40]. Consistent with the efficiency of BATD, and its ability to individualize treatment and promote treatment adherence, we conducted an open-label trial (*n* = 10) using a literal Spanish translation of the original BATD manual without global cultural modifications. Across the treatment course, depressive symptomatology decreased, and the proposed BATD mechanisms (activity engagement and environmental reward) increased. Further, activation simultaneously corresponded with decreases in depression: as activation increased, depression decreased. On the other hand, environmental reward predicted depressive symptomatology. Results indicated sustained clinical gains at the 1-month follow-up. Of note was that treatment adherence and attendance were high in this small sample; eight participants completed treatment. Taken together, BA but especially BATD appears to be a promising depression treatment among Latinos with LEP. The logical next step involves conducting an RCT.

**Figure 1:**
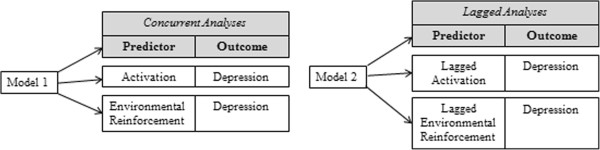
**Representation of models that will test the correspondence between increases in activation and contact with environmental reinforcement, and decreases in depression.** Model 1 will test the simultaneous correspondence between depression with activation and environmental reinforcement. Model 2 will test if increases in activation and contact with environmental reinforcement predict depression.

Following up this promising initial work, the current study is comprised of an RCT with three aims. First, we will compare the impact of BATD and a supportive counseling (SC) condition on participants’ depression throughout the course of the study. We expect that participants randomized to BATD will evidence greater reductions in depressive symptoms and a higher percentage remission of MDD relative to participants randomized to the SC condition. Second, we will compare group differences on the proposed mediators of BATD, including activation and contact with environmental reward. We hypothesize that participants assigned to the BATD condition will evidence greater increases in activation and contact with environmental reward relative to those assigned to the SC condition. We also expect that increases in activation and contact with environmental reinforcement will correspond with decreases in depression. Figure 1 is a depiction of the models tested under this aim. Third, we will examine SC and BATD group differences on key conceptually relevant variables associated with treatment retention and outcomes in Latinos, such as treatment satisfaction [41], therapeutic alliance [42], and perceived stigma [43], as these variables have been shown to affect treatment outcomes and retention in this population. In the context of the third aim, we will compare dropout rates between conditions. We expect that, relative to participants assigned to the SC condition, participants randomized to BATD will evidence higher treatment satisfaction given the heavy emphasis of BATD on putting effort into one’s recovery from depression ("poner de su parte"), a treatment expectation highly valued in this population [44,22]. Further, we hypothesize greater therapeutic alliance in the BATD condition given the collaborative approach expected to emerge between the client and the therapist in this treatment condition [45]. We also hypothesize that, as a result of the conceptualization of depression of BATD as originating from the lack of environmental reinforcement, perceived stigma levels will be lower relative to the SC condition. Finally, we expect that treatment retention will be greater for the BATD condition than for the SC condition based on previous findings suggesting this trend [39,40].

## Methods/design

### Design

Sixty depressed Latinos with LEP from the community will be randomized to receive individual BATD (*n* = 30) or an individual contact time-matched SC condition (*n* = 30). The design allows for the examination of treatment group differences in depression, activity level and contact with environmental reinforcement, treatment adherence, treatment satisfaction, therapeutic alliance, and perceived stigma between conditions, consistent with our three study aims. Assessments take place immediately prior to each session and at 1-month post-treatment. This study was approved by the University of Maryland-College Park’s Institutional Review Board (protocol no. 403879–2). We requested to obtain participants’ verbal informed consent and not written informed consent to protect the privacy and confidentiality of a sample that may be represented predominantly by immigrant Latinos, who may not be in the US on legal terms.

### Recruitment

Participants are being recruited from the District of Columbia Metro area, including Montgomery and Prince George’s counties in Maryland. Most participants are contacted through three different community organizations that serve predominantly low-income Latinos with LEP. Flyers are also posted in grocery stores, bus stops, public libraries, community centers, and consulates serving Latino populations.

### Eligibility

Initial eligibility is determined via a telephone screener, which includes questions from the Mood Disorders, Substance Use and Dependence, and Psychotic Disorders modules of the Structured Clinical Interview for the Diagnostic Statistical Manual version IV text revision (SCID-IV) [46]. To be included in the study, participants must: 1) be a minimum of 18 years of age; 2) be of Latino descent; 3) self-report LEP; 4) meet current MDD criteria; 5) have completed the 4^th^ grade of education or higher either in their country of origin or in the US; 6) not have current substance abuse or dependence; 7) have no bipolar or psychotic disorders; 9) not be currently receiving psychotherapy; and 10) if currently taking antidepressants, demonstrate pharmacological stability as indicated by 3 or more consecutive months of use. Excluded individuals are referred to mental health resources within the community.

### Procedure and randomization

After a participant is deemed eligible over the telephone, they are scheduled for a first appointment at the University of Maryland, College Park. At the beginning of the appointment, a research assistant reviews study procedures, answers the participant’s questions about the study, and obtains verbal informed consent. Along with the verbal informed consent, participants are made aware of the certificate of confidentiality obtained with the purpose of protecting any identifiable information and immigrant status from forced disclosure. Participants are also informed about randomization procedures. Specifically, participants are told that they have an equal chance of receiving one of two psychotherapies that have yet to be formally evaluated among Latinos with LEP. No details about the treatment content was provided. After providing verbal informed consent, a member of the staff trained in administering the SCID-IV will administer the Mood Disorders, Substance Use and Dependence, Psychotic Disorders, and Anxiety Disorder modules of the interview to confirm eligibility for the study and characterize the sample’s psychopathology appropriately.

**Figure 2:**
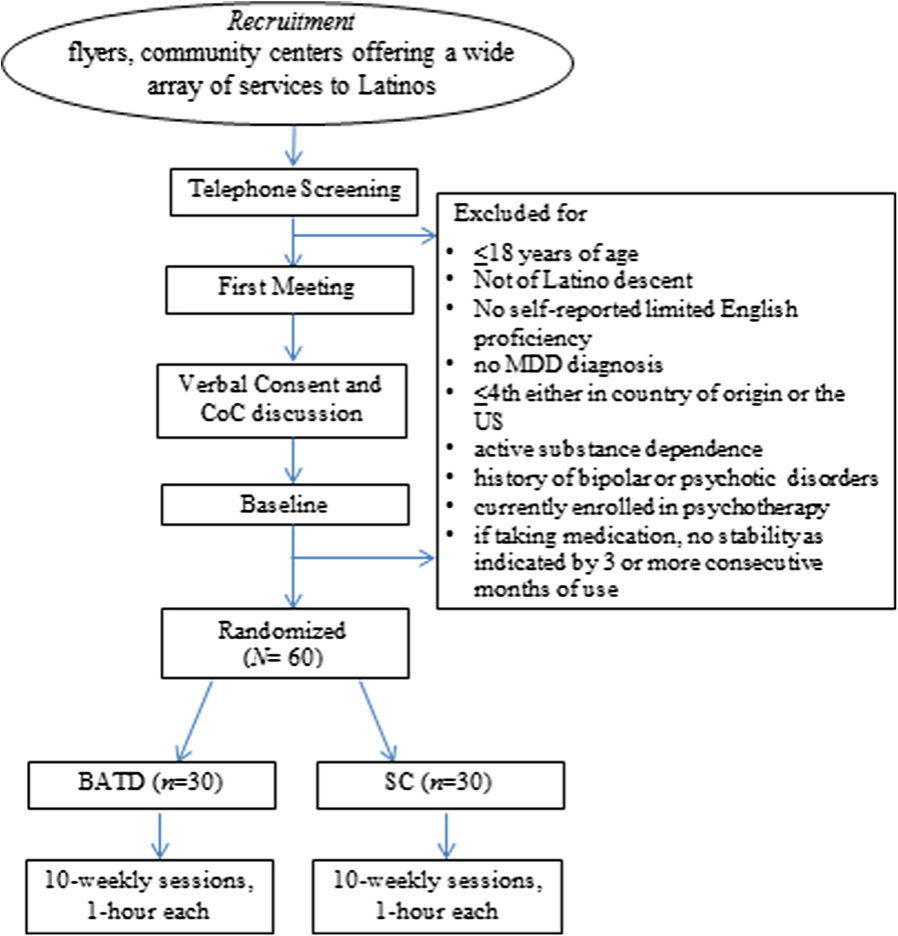
**Flow diagram from recruitment to randomization. BATD,** behavioral activation treatment for depression;** CoC; MDD,** major depressive disorder;** SC,** supportive counseling.

Participants earn $15 for the completion of questionnaires at the baseline assessment and the post-treatment assessment (corresponding to sessions 1 and 10 of treatment). Participants are paid $10 for the remaining scheduled assessment points. Participants are assessed prior to each treatment session. In the situation that participants do not qualify during the baseline interview, they are paid for completing the baseline assessments. In general, the completion of the assessments during the first and last meetings take up to 70 minutes, and up to 20 minutes for the remaining meetings. After completing the assessments, participants receive compensation.At the first meeting, a staff member not involved in the study conducts the randomization using a computerized random number generator and informs the participant’s therapist of the assigned therapy condition in person. The research assistant conducting assessments for a participant is blind to every participants’ assigned treatment condition. Blinding is facilitated by the fact that participants complete identical assessment measures regardless of their assigned condition throughout the course of the study. This removes any need for research assistants to be informed about participants’ randomization outcome. Therapy commences thereafter, and therapy sessions last approximately 60 minutes. Figure 2 provides a flow diagram of study procedures from recruitment to randomization.

### Therapists and research staff

Therapists for the proposed project are postbaccalaureate research assistants and graduate students with native fluency in Spanish (*n* = 6). Given the differing degrees of clinical training among the therapists, randomization across conditions takes place to control for therapist effects. Treatment integrity and fidelity is ensured through extensive therapist training and supervision. As training, therapists completed six, 2-hour workshops conducted by the BATD developer. They also attend 2 hours of ongoing weekly supervision with two doctoral level psychologists, including the treatment developer (CWL) and a second co-author (LM). During these supervisions all therapy cases are discussed. Further, therapists attend a 1-hour weekly supervision meeting in Spanish with the first and second authors (AC and KEL). Goals of supervision include ensuring BATD and SC treatment fidelity and standardization and discussing participant progress and challenges encountered at each session. Treatment fidelity strategies for monitoring and improving provider training procedures outlined by the National Institutes of Health Behavior Change Consortium [47] are followed.

For the purpose of supervision, therapy sessions are audiotaped with the permission of participants. Spanish-speaking research assistants review 20% of audiorecorded sessions to determine treatment adherence and fidelity within 1 week of the therapy session using separate rating checklists and scales developed for the SC and BATD conditions. Any treatment divergence is brought up with the respective therapist and is discussed during supervision meetings.

### Overview of treatments

#### ***Behavioral activation treatment for depression***

We are using the most current version of the Spanish BATD manual [26] for this trial. As outlined in the manual, the first session of BATD focuses on providing psychoeducation about depression, reviewing the treatment rationale, discussing the importance of monitoring daily activities, describing session attendance policies and stressing the importance of attending every session weekly, and explaining the relationship between treatment adherence and the likelihood of treatment success. Starting in the first session and continuing until the end of treatment, the homework assignment focuses on participants monitoring their daily activities until the subsequent session and reporting a numerical rating of both enjoyment and importance for each activity completed.

The second BATD session consists of briefly reviewing the content of the previous session, discussing activities completed as well as the ratings of enjoyment and importance, making use of the completed daily monitoring record forms, and assessing any difficulties associated with homework completion. The remainder of the second session is devoted to a thorough discussion of life areas (for example, relationships) and corresponding values (for example, be a caring husband) important for participants, with the purpose of selecting activities consistent with these values in future sessions (for example, taking spouse on monthly dates).

During the third session, participants work on selecting at least fifteen activities that they consider rewarding (taking into consideration both expected enjoyment and importance) that are consistent with life areas they deem important and their expressed personal values. These could constitute activities that are already a part of the participants’ schedule or new activities. Participants rank the activities in terms of difficulty. They complete easier activities toward the beginning of treatment and progress toward more challenging activities. During sessions 4 through 10, participants work toward accomplishing three to five activities on their list that reflect their values.

During session 5, participants are introduced to “contracts”, which provides the opportunity to request assistance from friends and family. Participants use contracts in order to ask for help with accomplishing selected activities that are challenging or that may be more “enjoyable” and “important” with company. No new material is introduced beyond this point. Sessions 6 through 10 consist of continued engagement in meaningful activities and daily monitoring, as well as discussions of an individualized post-treatment plan within a behavioral activation framework of scheduling activities corresponding to participants’ values and drafting “contracts” with people in their support network. Throughout treatment, depressogenic and non-depressogenic patterns are identified with the assistance of the monitoring forms and ratings of enjoyment and importance.

#### ***Supportive counseling***

To control for the non-specific elements of therapist contact, approximately 30 clients are randomized to receive SC. The SC manual used for the current study is modeled after Novalis and colleagues [48]. The rationale of SC is that depressed clients may benefit from sharing their feelings, thoughts, and/or experiences in order to relieve some of their emotional burden. This relief is facilitated by the therapist’s provision of a supportive, non-judgmental, and confidential environment. The opportunity to release emotional burden is consistent with some reports that suggest that Latinos tend to expect therapy to provide the opportunity for “desahogo” (that is, to get things off one’s chest) [49]. Focus groups with Latino clients have also described this treatment expectation [15]. The rationale for SC is particularly relevant for Latino immigrants, who may lack support in the US and whose LEP may prevent establishing new connections.

SC does not follow a specific theoretical model and is best described as offering the client unconditional support. The discussion for each session is patient-driven, and the manual includes training in therapy using SC techniques including reflections, empathic listening, encouragement, and help in exploration and expression of feelings and experiences, without advice giving, solution offering or skills teaching. Each SC session concludes with a scripted relaxation exercise. The manual is organized in a way that reviews the basis for supportive psychotherapy, the meaning of establishing a supportive relationship, steps for beginning the therapy session, session management, crisis management, and ethical factors in supportive psychotherapy. Therapists are trained in these procedures, in using non-directive techniques and in avoiding BATD techniques. These topics are covered in different chapters within the manual.

The SC condition also includes journal writing homework forms. The amount of homework assigned in the SC condition is matched with BATD. Participants are asked to write in the journal forms once a day about any topic that they would like.

#### ***Assessments***

In line with our study aims, participants complete questionnaires to assess five principal domains. Table 1 offers a summary of the questionnaires, the domain they assess, and the time-point at which they are administered.

**Table 1:** Schedule of questionnaire administration

**Assessment**	**Baseline/Session 1**	**Session 2**	**Session 3**	**Session 4**	**Session 5**	**Session 6**	**Session 7**	**Session 8**	**Session 9**	**Session 10**	**1-month post-treatment**
Domain 1: participant characteristics											
Demographics	X										
Acculturative stress	X										
Other medication/treatment use	X									X	X
Domain 2: depressive symptomatology											
SCID-IV modules	X									X	X
Beck Depression Inventory-II	X	X	X	X	X	X	X	X	X	X	X
Hamilton Rating Scale for Depression	X	X	X	X	X	X	X	X	X	X	X
Domain 3: behavioral activation and reinforcement derived from the environment											
Behavioral Activation for Depression Scale	X	X	X	X	X	X	X	X	X	X	X
Reward probability index	X	X	X	X	X	X	X	X	X	X	X
Domain 5: attitudes toward treatment											
Stigma checklist	X				X					X	X
Therapeutic alliance	X				X					X	X
Treatment satisfaction	X				X					X	X

SCID-IV, Structured Clinical Interview for the Diagnostic Statistical Manual version IV text revision.

##### 

**Domain 1 - participant characteristics** A standard demographics questionnaire assesses participants’ education, income, years of residence in the US, depression treatment history, immigration status, and reason(s) for immigrating (if applicable).

The Multidimensional Acculturative Stress Inventory [50] was originally created to measure acculturative stress from living in the US for individuals of Mexican origin; the scale is comprised of four subscales including English competency pressures (7 items), pressure to acculturate (7 items), pressure against acculturation (4 items), and Spanish competency pressures (7 items). Given the non-applicability of the last subscale for the current sample, we will only use the first three subscales. Higher scores indicate greater stress. The Spanish version of the questionnaire has achieved Cronbach alpha values ranging from 0.74 to 0.91.

To determine study eligibility and the potential effect of pharmacotherapy or other medications on the results of the treatment, we collect information on participants’ medication use, including the names of medications and length of use. Participants are excluded from the study if they report taking medication but do not demonstrate psychotropic stability as indicated by 3 or more months of consistent use.

##### 

**Domain 2 - depressive symptomatology** To identify depressive mood variations through the study trial, we utilize the Beck Depression Inventory (BDI)-II [51]. The inventory consists of 21 items that assess severity of depressive symptomatology. BDI cumulative scores range between 0 and 63; scores ranging between 14 and 19 are indicative of mild depression, scores between 20 and 28 are indicative of moderate depression, and scores of 29 or above are indicative of severe depression. The Spanish version of the BDI-II was developed by Sanz and colleagues [52] and was evaluated with a sample of 470 Spanish community adults.

The Hamilton Rating Scale for Depression (HRSD) [53] is used as a clinician assessment of depressive symptom severity. It is administered weekly in accordance with standard practice in clinical trials and clinical practice [54]. This measure is a 17-item scale including questions pertaining to libido, energy, weight, and appetite changes. Scores are combined into a single total score, and the measure has been shown to have strong divergent/convergent validity and reliability. The psychometric properties of the Spanish version of the HRSD have been investigated in two studies [55,56] in which it demonstrated acceptable internal consistency (0.72), good inter-rater reliability (0.99), and good split-half reliability (0.89).

Additionally, to establish an MDD diagnosis and evaluate remission rates, we administer the SCID-IV [46]. For the current study, specific modules of the SCID-IV are used to assess for: 1) primary affective disorders, including major depression and manic episodes; 2) substance use disorders, including abuse and dependence; 3) primary anxiety disorders, including panic disorder, generalized anxiety disorder, and post-traumatic stress disorder; and 4) and psychotic symptoms.

In the case that participants report intent to harm themselves, we administer the Modified Scale for Suicidal Ideation (MSSI) [57]. The questionnaire consists of a semi-structured interview comprised of 18 items that assess suicidal thoughts or behaviors over the past 2 days. The questionnaire contains items related to suicide intent, plan, and means as well as desire and reasons to live or die. Each item is scored from 0 to 3, with higher numbers indicating higher risk. The scale is only administered as needed. The MSSI was translated to Spanish for the initial pilot study [32] to assess for severity of suicidal ideation. Please see the 'Protection of Participants' section below for more details on the use of this measure in the present study.

##### 

**Domain 3 - behavioral activation and reinforcement/punishment derived from the environment** We are utilizing two different measures of activation in our study given purported differences between the constructs they are intended to assess: the Behavioral Activation for Depression Scale (BADS) [58] and the Reward Probability Index (RPI) [59].

BADS consists of 25 items and was designed to measure the extent to which individuals become more activated and less avoidant through the course of the BA intervention. The measure includes four subscales: activation, avoidance/rumination, work/school impairment, and social impairment. Given that examining participants’ activation levels throughout the treatment course is highly relevant to our study hypotheses, we will examine increases in the total BADS scale as well as in the BADS activation subscale specifically. The activation subscale contains items related to the engagement in focused, goal-directed activities as well as to the completion of scheduled activities [60] which allows examining activation changes while isolating impairment elicited by avoidance or rumination (also measured within the BADS). Items comprising this subscale include “I am content with the amount and types of things I did” and “I engaged in a wide and diverse array of activities”. The internal consistency of the Spanish version of the complete BADS scale has been reported at 0.80, and at 0.81 for the BADS activation subscale when administered to a sample comprised of students at a Spanish university [60].

The RPI is a 20-item scale that was developed to assess availability of reinforcement in the environment. The total RPI consists of two subscales: 1) the reward probability index, which includes items measuring the likelihood that individuals are able to obtain reinforcement through instrumental behaviors; and 2) the environmental suppressors index, which includes items that describe the presence of aversive and unpleasant experiences in the respondents’ environment [59]. Total RPI score is calculated by adding scores of the items measuring reward probability index with reversed scores of the items measuring environmental suppressors. Internal consistency of the total RPI scale was *α* = 0.90 and the test-retest reliability was *r =* 0.69 in the original validation study [59]. Because there is no psychometric evaluation of a Spanish translation of the RPI, the team that translated the original BATD treatment into Spanish also translated this assessment tool. AC was responsible for back-translating the items into English (please see [61] for more information about this procedure). Discrepancies between the back-translation and the original version of the questionnaire were discussed among the parties and addressed. In the original validation study [60], psychometric properties of each subscale suggested a strong internal consistency (*α* = 0.82 to 0.90) as well as strong test-retest reliability (*r* = 0.83 to 0.86) [59]. In the preliminary study conducted by Collado and colleagues [32], the Cronbach’s alpha for the Spanish translation of the total RPI scale ranged between 0.83 and 0.89. Further, Cronbach’s alpha for the reward probability index subscale ranged between 0.84 and 0.95, and between 0.73 and 0.88 for the environmental suppressors index subscale across sessions, indicating strong levels of internal consistency.

##### 

**Domain 4 - treatment adherence** Session attendance is logged for every client. In addition, we will calculate homework completion for each client by dividing the total number of completed homework sessions by the total number of assigned homework sessions.

##### 

**Domain 5 - treatment correlates** To measure stigma-related concerns associated with receiving treatment for depression, participants complete the Stigma Checklist Questionnaire [62], a measure specifically designed for use with low-income Spanish-speaking or bilingual Latino patients. The questionnaire consists of 7 items designed to identify participants’ perceptions of others who have depression and take medication, as well as their fear of relatives learning that they are dealing with depression. The reliability of the scale has been reported at a Cronbach’s alpha of 0.69.

The Therapeutic Alliance with Clinician Questionnaire [63] assesses the strength of the therapeutic relationship using a 9-item Likert scale format. The Spanish version of the questionnaire [64] was evaluated with a sample predominantly comprised of depressed individuals and achieved high internal consistency (*α* = 0.96) and an item component correlation of 0.70. The authors concluded that the measure has both clinical and research utility.

To elicit feedback about both treatment conditions, we administer an in-house developed questionnaire every other session. Participants rate the treatments on a 0 to 5 Likert-type scale across 10 items including “to what extent do you believe that this treatment has improved your depression/low mood?”, and “how valuable do you think this treatment would be for individuals who experience depressed/low mood?” This treatment satisfaction scale ranges from 0 to 50, with higher scores representing higher satisfaction.

### Protection of participants

Given that a diagnosis of MDD is a criterion for eligibility, participants’ safety and privacy throughout the course of the study is a priority. Accordingly, we have incorporated a Data Safety Monitoring Board (DSMB) and a detailed safety protocol. The DSMB was created to monitor and ensure participants’ safety through the course of treatment. Members of the DSMB include five independent researchers who have extensive experience in treatment research with BATD and depression treatment research in general. These individuals are involved in the project as DSMB members in order to guarantee objectivity regarding participants’ safety, study conduct, and recommendations concerning the continuation or modification of the project’s safety protocol. These members participate in an annual teleconference to conduct continuous evaluation of the clients’ safety protocol. In addition, monthly reports regarding clients’ progress in both treatment conditions are generated. Any potential issues that pose risk to participants (during screening or during the study course) are discussed over the telephone with the DSMB.

For immediate crisis intervention, we developed a thorough safety protocol. Every research assistant and therapist involved in the study is trained in these procedures following already established protocols for depression treatment development projects conducted at the laboratory. In the case that a participant reports suicidal intent and a plan, research assistants administer the MSSI [57]. Prior to administering the MSSI, the research assistant notifies the authors involved in the study. Immediately after conducting the MSSI, the research assistant informs the participant’s therapist of the results. The therapist engages in an honest discussion with the participant about his or her likelihood of carrying out suicide. The therapist conducts a lethality risk assessment and draws a contract with the participant indicating that he or she will not make any attempts to carry out the plans. The therapist and research assistant document the outcome of the discussion and their impressions. The appropriate authorities are notified if the participant is perceived to be in imminent danger (in or out of session).

Within 24 hours of conducting a safety protocol, a conference call discussing relevant details takes place with all of the DSMB members. The goal of this discussion is for DSMB members to provide a future plan to ensure the participant’s safety. A similar protocol is followed in the case that a participant reports suicidal ideation to his or her therapist. In that case, the MSSI is not administered; rather a lethality risk assessment is given primary emphasis. All other procedures are then followed as described above.

### Sample size considerations

We based our sample size needs on effects observed in the literature, including other treatment studies, and by making informed decisions about the magnitude of effects of BATD in this population that would likely be of clinical significance. For the primary hypothesis that BATD would result in greater reductions in depressive symptoms, we calculated the sample size required based on a recent meta-analysis conducted that examined the effect size of BA relative to control conditions [29] and on a treatment study that compared cognitive behavioral therapy to an active treatment condition (interpersonal psychotherapy) conducted by Rosselló and colleagues [65] in a Spanish-speaking Latino sample. These studies yielded effect sizes of 0.43 and 0.74, respectively, indicating medium to large effects. Our proposed sample size of 30 in each cell fits well within that suggested by a power analysis using these two reported effect sizes to allow for a power of 0.80 using an alpha of 0.05 [66]. Our sample size also follows recommendations of including 15 to 30 subjects per cell in this stage of treatment development [67].

### Data analysis plan

We will first assess equivalence of random assignment of groups, patterns of missing data, research dropout rates, and distributional properties of all measures. Hierarchical linear modeling (HLM) [68] will be used to examine within-subject change of depression (first aim) and activation and reinforcement derived from the environment (second aim) over the course of treatment. The nature of HLM analyses allows us to control for baseline scores of each measure, include multiple measurement points while accommodating missing data, examine individual change over time in outcomes, and include the average change and the individual variation around this average change. We will specify all of our HLM level-1 intercepts and slopes as random, given that we expect first session depressive symptomatology, activation, and availability of reinforcement in the environment, as well as each individual’s slope of these constructs, to differ across our participants. We will center all variables of interest around the mean of respective scores to avoid multicollinearity. Planned covariates in these analyses will include gender, recruitment method, and baseline levels of depressive symptoms, as well as the main effect of treatment condition and the linear effect of time. Inclusion of the time by treatment condition interaction will indicate the extent to which treatment differences are more or less pronounced early versus later across treatment sessions. Categorical rates of remission of MDD will be examined across treatment conditions using the Cochran-Mantel-Haenszel test, controlling for gender.To examine the correspondence between increases in activation and environmental reinforcement with depression, we will also use HLM to examine correspondence of these variables over time. These models will include the aforementioned covariates in addition to including activation and environmental reinforcement as time-varying predictors of depression, the main effect of treatment condition, the linear effect of time, and the inclusion of time by treatment condition interaction. Because of the difficulty in attributing causality between variables assessed at the same time-point, we will also conduct analyses predicting depression with environmental reinforcement and activation assessed at the previous time-point as time-varying covariates (please see Figure 1 depicting the lagged and concurrent analyses). These lagged analyses will indicate whether the predictors predict change in depression at subsequent assessments, consistent with the BATD framework that activation and environmental reinforcement are expected to increase before depression decreases.

To examine more closely the effects of treatment on attendance, we will conduct a Cox proportional hazards survival analysis predicting days to attrition. This will allow us to examine the extent to which BATD was able to increase the latency to treatment attrition. Homework completion will be assessed by dividing the total number of completed homework sessions by the total number of assigned homework sessions for each participant. We will conduct *t*-tests to examine differences in homework completion between BATD and SC.

Finally, we will conduct *t*-tests examining differences between BATD and SC in treatment satisfaction, therapeutic alliance, and perceived stigma at each session these correlates were examined.

## Discussion

The current study is an RCT of 60 depressed Latinos with LEP in the community randomized to BATD (*n* = 30) or to SC (*n* = 30). Strengths of the trial involve a contact time-matched control and the utilization of MDD as inclusion criteria rather than elevated depressive symptomatology. Another important aspect of the study is that, in comparison to other treatment trials among Latinos, the current study is utilizing a direct, Spanish translation of the BATD manual without *a priori* cultural modifications. Similar to the open-label trial, we made this decision because BATD facilitates treatment customization for each client. Therefore, throughout treatment, the therapist emphasizes only values that are important to the client without prior assumptions about their culture.

We also foresee specific study limitations. First, given that the current study is being conducted as part of a small, pre-doctoral grant, funds are limited for recruitment in mass media, limiting the reach of our efforts. In addition, we may observe a high dropout rate in this study, consistent with trends in treatment development studies with this population [16,17]. Given that our sample is already small, small but significant differences between the treatments may go undetected (type II error). However, this concern is mitigated given the important information that may be gained through the study, including the efficacy of the two therapies tested, the proposed mechanisms of change, and the treatment correlates examined. Another limitation is the high rates of comorbid psychopathology we may observe in our sample. This would be consistent with previous literature in this particular population and our observations from the BATD open-label trial. Although we believe that this fosters external validity of our results in the context of an RCT, it may also result in decreased overall treatment gains [69].

To our knowledge, this constitutes the first effort towards conducting an RCT comparing a behavioral intervention to SC with a sample of depressed Spanish-speaking Latinos in the US with LEP. This group has been historically under-represented in both clinical and research samples [70,71], which has prevented drawing conclusions about the efficacy of psychotherapeutic treatments for depression for this population. Together, the increasing Latino with LEP population, the elevated MDD rates among Latinos with LEP, high attrition rates, and suboptimal treatment gains reported in previous depression treatment studies, make the evaluation of BATD in this group a pressing need. Therefore, the proposed study will not only contribute to a scarce yet much-needed evidence base, but it will also set the stage for a larger RCT. Larger RCT studies may be able to incorporate standard of care treatments in addition to make possible the examination of BATD-treatment mechanisms.

## Trial status

Currently recruiting.

## Abbreviations

BA: behavioral activation; BADS: Behavioral Activation for Depression Scale; BATD: behavioral activation treatment for depression; BDI: Beck Depression Inventory; DSMB: Data Safety Monitoring Board; HLM: hierarchical linear modelling; HRSD: Hamilton Rating Scale for Depression; LEP: limited English language proficiency; MDD: major depressive disorder; MSSI: Modified Scale for Suicidal Ideation; RCT: randomized controlled trial; RPI: Reward Probability IndexSC supportive counseling; SCID-IV: Structured Clinical Interview for the Diagnostic Statistical Manual version IV text revision.

## Competing interests

The authors declare that they have no competing interests.

## Authors’ contributions

AC, CWL, and LM conceived and designed the study. AC executed the study. CWL developed the intervention. AC and KEL drafted the manuscript and serve as peer clinical supervisors to the therapy team. AC and KEL edited the final manuscript. LM and CWL oversaw study implementation and were closely involved with all stages of manuscript preparation. All authors read and approved the final manuscript.
